# Japanese Quail’s Genetic Background Modulates Effects of Chronic Stress on Emotional Reactivity but Not Spatial Learning

**DOI:** 10.1371/journal.pone.0047475

**Published:** 2012-10-11

**Authors:** Agathe Laurence, Cécilia Houdelier, Christophe Petton, Ludovic Calandreau, Cécile Arnould, Angélique Favreau-Peigné, Christine Leterrier, Alain Boissy, Marie-Annick Richard-Yris, Sophie Lumineau

**Affiliations:** 1 Ethos UMR 6552, Université de Rennes 1, CNRS, Rennes, France; 2 UMR 85 Physiologie de la Reproduction et des Comportements, INRA, CNRS, Université de Tours, Nouzilly, France; 3 Unité de Recherche sur les Herbivores, INRA, St Genès Champanelle, France; 4 UMR791 Modélisation systémique appliquée aux ruminants, INRA, Paris, France; Liverpool John Moores University, United Kingdom

## Abstract

Chronic stress is known to enhance mammals’ emotional reactivity and alters several of their cognitive functions, especially spatial learning. Few studies have investigated such effects in birds. We investigated the impact of a two-week stress on Japanese quail’s emotional reactivity and spatial learning. Quail is an avian model widely used in laboratory studies and for extrapolation of data to other poultry species. As sensitivity to chronic stress can be modulated by intrinsic factors, we tested juvenile female Japanese quail from three lines, two of them divergently selected on tonic immobility duration, an indicator of general fearfulness. The different emotional reactivity levels of quail belonging to these lines can be revealed by a large variety of tests. Half of the birds were submitted to repeated unpredictable aversive events for two weeks, whereas the other half were left undisturbed. After this procedure, two tests (open field and emergence tests) evaluated the emotional reactivity of treated and control quails. They were then trained in a T-maze for seven days and their spatial learning was tested. The chronic stress protocol had an impact on resting, preening and foraging in the home cage. As predicted, the emotional reactivity of treated quails, especially those selected for long tonic immobility duration, was higher. Our spatial learning data showed that the treatment enhanced acquisition but not memorization. However, intrinsic fearfulness did not seem to interact with the treatment in this test. According to an inverted U-shaped relationship between stress and cognition, chronic stress can improve the adaptability of birds to a stressful environment. We discussed the mechanisms possibly implied in the increase of emotional reactivity and spatial abilities.

## Introduction

Whether natural or experimental, chronic stress has numerous influences on the physiology and behaviour in various species [1], [2]. In experimental conditions, chronic stress is defined as a prolonged period of stress during which an individual is exposed to continuous or repeated psychological stressor(s) without habituation, by unpredictable administration of different types of intermittent mild stressors [3]. Chronic stress can affect growth, hypothalamus-pituitary-adrenal (HPA) axis response and emotional reactivity, and can induce several brain and cognitive alterations [4]–[6]. However, analysis of the numerous studies in this field shows that chronic stress effects are protocol dependent, especially in regard to the intensity and the duration of the stressors used [7]–[9]. In mammal studies, chronic stress procedures aim to induce long-term and marked effects such as anhedonia used as models in the etiology of human depression [10]–[13]. By contrast, chronic stress effects on the behaviours of other taxa are scarce (amphibians: [14], fish: [15], [16]) and then procedures seem to be less rigorous than those used for rodents. Whereas reports show that chronic stress procedures can influence birds’ physiological parameters (Starlings (*Sturnus vulgaris*: [3], [17]–[19], Japanese quail (Coturnix c. Japonica): [20]) very few studies have investigated behavioural changes [21], [22]. Other avian studies using artificially induced postnatal stress (e.g. corticosterone feeding or injections) evidenced HPA axis modifications and enhanced emotional reactivity or impaired cognitive performance [23]–[25]. However, previous studies on starlings, zebra finches (*Taeniopygia guttata*) and Japanese quail report a decrease of basal corticosterone levels after exposure to unpredictable and repetitive mild stressors [3], [17], [22] and chronically stressed birds’ cognitive abilities vary between studies [21], [26]. Thus, regardless of the species studied, these results raised the possibility of an inverted U-shaped relationship between stress intensity and cognitive functions [27].

Another source of variability of the effects of chronic stress procedures could be inter-individual variability of the sensitivity to chronic stress procedures [28]–[31]. Indeed, the more emotive an individual is the more sensitive to environmental stressors it might be. Japanese quail, a terrestrial bird used in the laboratory and poultry stock breeding have been selected for either low or high emotional reactivity [32]. In fact, tonic immobility (TI), a reflexive antipredator response correlated to general fearfulness, varies greatly in duration [33]. Several traits of the fear responses of Japanese quail selected for either short or long TI durations (respectively STI and LTI) diverge [34]–[38]. These two selected lines of Japanese quail were used in this study to test the impact of intrinsic emotional reactivity on the sensitivity to a two-week chronic stress procedure. As birds’ chronic stress has been poorly studied, we first assessed how spontaneous behaviour in their home cage was affected by our procedure and estimated its possible modulation by intrinsic fearfulness. Then, two major aspects of chronic stress effects were investigated: emotional reactivity in a novel environment (open or closed) and spatial learning. Chronic stress increases emotional reactivity and anxiety [13]. Moreover, fearful individuals are more sensitive to changes in their environment [39]. Thus, we hypothesized that sensitivity to chronic stress procedure depends on intrinsic fearfulness, then LTI treated quail should be the most affected by a novel environment and that STI quail (the least fearful) would be less affected by our procedure than LTI quail (the most fearful). Quail which are the most sensitive about chronic stress should be more reactive in novel environment, depending on the situation. Previous studies report that differences in fearfulness depend on several factors, as the device characteristics (e.g. open or closed, with or without dust-bathing substrate), or the controllability of the situation (e.g. to enter freely or not in a novel environment) [40], and several authors highlight the multidimensional aspect of fear in birds and other taxa [2], [38]. In addition, emotional reactivity plays a major role in organisms’ capacities to adapt to their environment as it can influence several behavioural characteristics including cognitive processes [41]–[43]. Indeed, several cognitive mechanisms like perception, attention or memory storage can be affected by the emotional state [9], [42]. We are the first to investigate spatial learning abilities in Japanese quail selected on their tonic immobility duration. We hypothesized that emotive animals should be more attentive to their environment [39] and thus would have better performance. Thus, differences in emotional reactivity between genetic lines could subsequently modulate cognitive performance in a spatial learning task, especially after exposure to chronic stress which should enhance differences between these lines. Nonetheless, depending on the mechanisms involved in the cognitive process, the effects of chronic stress procedures seem to affect individual performances differently [21]. We tested two dimensions of spatial learning in a T-maze: acquisition and memorization. Currently, the effects of chronic stress on the mammals’ hippocampus are well studied [44], [45] and this brain structure plays a similar role in birds [46].

## Methods

### Ethics Statement

Animal care procedures were conducted in accordance with the guidelines set by the European Communities Council Directive (86/609/EEC) and French legislation on animal research. Our protocol was approved by the regional ethic committee (CREEA: “*Comité Rennais d’Ethique en Expérimentation Animale”* meaning “Rennes city Ethics Committee in Animal Experimentation”) (agreement n°R-2011-SLU-01).

### Animals and Housing Conditions

Our subjects were 88 female Japanese quail (*Coturnix coturnix japonica*). Eggs from two genetic selected lines from the 43^rd^ generation (STI and LTI: short/long tonic immobility duration) and from a control line (CTI) were provided by the *Pôle d’Expérimentation Avicole de Tours* (UE PEAT, INRA, Nouzilly, France). They were incubated for 17 days in a collective incubator (37°C, 45% humidity) at our laboratory. Details of the selection procedure based on the duration of tonic immobility (TI) have been described by Mills and Faure [35]. STI quail have very short tonic immobility durations whereas LTI quail have long tonic immobility durations (mean durations of tonic immobility at the 40^th^ generation (±SEM), STI line: 14 s (±35); LTI line: 228 s (±80)) [38]. CTI quail are not selected and present intermediate tonic immobility durations.

After hatching, subjects of each line were reared separately, in groups of 30 chicks. Cages (93×45×32 cm) were provided with two heating lamps (38±1°C). During all the experiment, quail were reared under a 12∶12 light-dark cycle and ambient temperature of 19±2°C; water and food were supplied *ad libitum*. During the first 2 weeks after hatching, the rooms were dimly lit at night (30 lux) with a green light in order to help the chicks find heat, without influencing their sexual development [47].

When they were 17 days old, their sex was determined by feather dimorphism and only females were kept for this study. They were wing-tagged and transferred to individual cages (24.5×35×18 cm) in four-tier batteries. Each tier contained four quails of a same genetic line (they were able to see one another as they shared a feeder), and each battery contained quails from the three genetic lines. Batteries were in four similar rooms.

### Chronic Stress Protocol (CSP)

To induce chronic stress, subjects from the treated group were exposed to repeated aversive events (stressors) two to four times a day, repeatedly for two weeks. The stressors could occur at any time during daytime or the dark phase; they were applied randomly and varied in duration (from 2 to 180 min). This reduced the possibility of habituation to the stressors and unpredictability is known to enhance domestic fowl’s (*Gallus gallus*) stress reactions [48]. The stressors used were adapted from studies of rodents [49] and birds [17], [19], [50] and are known to induce fear-related responses in the Japanese quail [21], [22]. Five of the stressors used were applied individually: a metallic stick banged on the cage rods twice in 2 min (noise and suddenness), a novel object waved in the cage (novelty and suddenness) once in each cage, air or water spouted twice in 30 min, and physical restraint in a home cage corner for 30 min. Four other stressors used during the CSP were applied to the entire battery: it was rocked for 2 min, three times in 30 min, ventilators switched on automatically and repeatedly for 5 to 15 min during the night, access to food was delayed by placing transparent devices on the troughs just before daylight for 3 hours, and unexpected sounds lasting 4 seconds were broadcast three times in 2 to 10 min, night and/or day. This last stressor consisted of three different sounds that had no biological signification for quail. CSP began when females were transferred to batteries (17 days post-hatching) and lasted for 14 days (31 day post-hatching). It was applied to half the birds (2 rooms) (treated group: N_STI_ = 18, N_CTI_ = 11, N_LTI_ = 18), whereas the other half (the other 2 rooms) (control group: N_STI_ = 17, N_CTI_ = 12, N_LTI_ = 12) was left undisturbed, except for the experimenter’s regular visits.

Quail were never manipulated directly by the experimenter except for physical measurements. When stressors were applied, the experimenter was dressed in white (gown) and blue (mask, gloves, and shoes), otherwise he was dressed in green (i.e. measures and care).

### Quail’s Activity in their Homecage

Time budgets were evaluated every day between the 9^th^ and the 14^th^ day of the CSP. The experimenter dressed in a green outfit stood upright and motionless in front of the open rooms for 2 h, when no stimulations were applied to treated quail. Quail’s activity was recorded using the instantaneous scan sampling with an 8 min interval, and the behavioural activities recorded were: resting (crouching and eyes closed), comfort behaviours (i.e. preening, stretching/scratching body parts) and food-directed behaviours (feeding and foraging in the trough). These activities have been used previously to estimate chronic stress in animals in their familiar environment [51].

### Emotional Reactivity Tests

At the end of the CSP (31-day old quail), chronic stress effects on emotional reactivity were assessed by two classical ethological tests for poultry each revealing a different aspect of this behavioural trait.

#### A. Emergence test

This test took place on day CSP+3. We followed a protocol similar to that described by Mills and Faure [52]. The subject was taken out of its cage and was put in a small dark wooden box (emergence box) (18×18×18 cm) that was then placed at the entrance of a large brightly lit cage (62×60×33 cm) with a glass front, wood shavings on the floor, and top closed. First, the emergence box remained closed for 1 min. During this time, the latency of the first contact call and the number of these calls were recorded. Then the emergence box was opened and the quail was allowed 3 min to emerge into the large cage. Latency to leave the box (head, first foot, and entire body) and latency of the first contact call were recorded. Once the quail was in the larger cage, a 3 min focal sampling recorded all its activities. The following parameters were recorded: locomotor and vocalizations latencies, number of vocalizations, fear activities (i.e. postures, freezing, trembling, running and jumping) and comfort activities (preening, scratching litter, dust bathing). Latency to leave the box is related to emotional reactivity: the more emotional an individual is, the longer it takes to leave the box [53], [54]. The different types of behaviours observed in the large cage are also indicators of emotional reactivity level (i.e. preening and dust bathing as positive markers and fear postures and escape attempts as negative markers). Finally, latency to call conspecifics and number of calls are indicators of social reinstatement, but they are modulated by fear of the novel environment, which inhibits vocal activity [55], [56].

#### B. Open field test

This test took place on day CSP+6. A quail was placed individually in the centre of a cylindrical arena (ø120 cm, H70 cm) with a linoleum floor, in the dark at first. Then, the light was switched on and all activities were recorded for 5 min. As for the emergence tests, first call and first step latencies were recorded. The number of steps was recorded, as well as exploration (pecking the walls and the floor) and fear behaviour (low observation posture, freezing, shivering). General activity in an open-field is known to be inversely correlated with emotional reactivity. Indeed, animals that are silent and stay still are considered more emotional than subjects presenting high vocal activity and exploration in this novel environment [57].

### Spatial Learning Test

Quail’s spatial learning capacities and memory were tested in a T-maze with beacons, 2 weeks after the CSP. They were given one training trial per day for six days. Then, seven days later, all quails were submitted to a supplementary training trial (retrieval test). To enhance their motivation in the maze, they were deprived of food 7 hours before each trial. The device was 20 cm wide. The main corridor was 80 cm long and the transversal corridor with one trough at the end of each arm was 100 cm long. At the end of one arm, a trough containing food was signalled by a black and pink stripped cue on the wall of the device. An identical but empty trough was placed at the end of the other arm, and signalled by a black disk on pink cardboard. Preference and avoidance for these cues were tested in a preliminary study and quail showed no significant preference for either of these two cues (time spent near the stripped cue, control quail: 57.4±7.1%, treated quail: 60.3±8.1%, Student *t* test compared to chance, control quail: *t* = 1.21, *P* = 0.25, *df* = 11; treated quail: *t* = 0.99, *P* = 0.34, *df* = 11). As in the emergence test, a test quail was put in a small dark box placed at the entrance of the maze. When this box was opened, the quail had to find the trough with food, always located on the same side for a given subject and latency to reach this trough was recorded. When the subject reached the arm with the reward trough first, this was considered a success, if not, an error was recorded. Quail were allowed 10 min to emerge from the box and to find the food, and a trial was ended when they had found the food and had eaten for 30 s.

### Statistical Analysis

Kolmogorov–Smirnov tests determined whether data residuals were normally distributed. Activity rates were transformed with arcsine square root transformation to fit a normal distribution and then two-way ANOVAs (line x treatment) and subsequent post-hoc Fisher LSD tests with Bonferroni corrections analysed data. When the interaction terms were not statistically significant, the analysis was rerun without them. Residuals for emotional reactivity tests data were not normally distributed, so Kruskal-Wallis and post-hoc Mann–Whitney U-tests with Bonferroni corrections were applied to compare control and treated groups for each genetic line.

Comfort behaviours were rarely observed in the open field test and were then analysed by Fisher’s tests on the numbers of quail in each group that expressed or not a given behaviour.

Spatial learning was analysed in two blocks: the first block (trials 1 to 3) and the second block (trials 4 to 6), using a repeated ANOVA design for binary data [58] within each block. The mean success rates for each group were compared to chance using Student t test. Finally, latency data to reach the food had normally distributed residuals after log-transformation and were analysed by an ANOVA and post-hoc LSD fisher tests.

Data are represented as means ± standard error of the mean (SEM). All analyses were performed using Statview Software (SAS, Cary, NC) with significance set at *P*≤0.05 except for post-hoc tests, with Bonferroni corrections.

## Results

### Quail’s Activity in their Homecage

**Figure 1:**
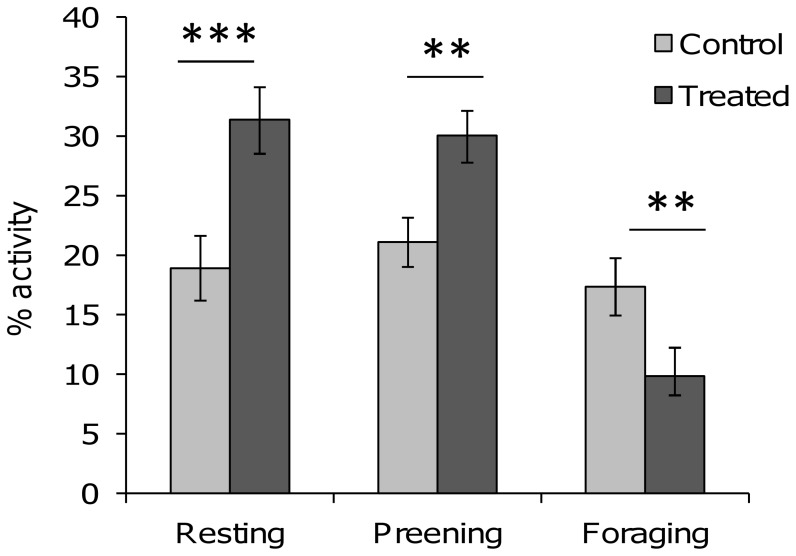
Activity (resting, preening and foraging) of control and treated quail in their home cage, at the end of the stress procedure (in %, means ±SEM). ANOVA, **P<0.01; ***P<0.001.

At the end of the CSP, treated and control quail’s activity rates differed significantly. Treated quail rested and preened significantly more but foraged significantly less than did control quail, (ANOVA, resting: *F*_[*1*], [*81*]_ = 12.38, *P*<0.001; preening: *F*_[*1*], [*81*]_ = 8.33, *P = *0.005; foraging: *F*_[*1*], [*81*]_ = 10.04, P = 0.002,; fig. 1).

### Emotional Reactivity Tests

#### A. Emergence test

Kruskal-Wallis tests revealed differences between groups for the number of conspecific calls emitted in the emergence box (*H*_[5,86]_ = 39.0, p<0.0001). However, treatment only affected this parameter in CTI quail: treated quail emitted more calls than did control quail (Mann**-**Whitney U-test, CTI, treated group: 18.2±1.4, control group: 3.1±1.4 calls, *U* = 23.0, *P* = 0.007; STI, treated group: 24.4±3.7, control group: 21.3±2.5, *U* = 149.0, *P* = 0.91; LTI, treated group: 6.1±2.0, control group: 4.3±2.1, *U* = 85.5, *P* = 0.63).

Only one female (control group, CTI line) did not emerge after the box had been opened for 3 min. This female was not included in further analyses. After quail had emerged, the number of shivering behaviours differed significantly between groups (*H*_[5,87]_ = 22.0, p<0.0001). Treated female CTI quail shivered significantly more frequently during the test than did control CTI quail (respectively 0.1±0.1 bouts and 0.9±0.2 bouts, Mann-Whitney U-test, *U* = 21.0, *P* = 0.003). We could evidence no significant differences between control and treated STI and LTI quail (STI, treated group: 0.1±0.1, control group: 0.2±0.2, *U* = 142.5, *P* = 0.73; LTI, treated group: 0.7±0.2, control group: 0.7±0.3, *U* = 104, *P* = 0.88).

####  Open field test

Treatment affected exploration of the floor (Kruskal-Wallis test: *H*_[5,87]_ = 18.2, *P* = 0.002) : treated LTI quail explored significantly less than did control LTI quail (number of explorations bouts, control quail: 11.8±1.5; treated quail: 4.9±0.9, Mann-Whitney U-test, U = 38.5, p = 0.002) but there was no effect of the treatment in the other genetic lines (STI, control quail: 13.3±2.2, treated quail: 15.0±2.9, *U* = 105.5, *P* = 0.95; CTI: control quail: 8.4±1.6, treated group: 13.6±2.1, *U* = 36.0, *P* = 0.07). Finally, LTI quail’s comfort behaviours were also significantly influenced by treatment: whereas 42% of the control quail scratched the linoleum floor and 50% dust-bathed, none of the treated quail expressed these comfort behaviours (Fisher LSD tests, scratching: *P* = 0.006; dust bathing: *P* = 0.002). Treatment affected neither STI nor CTI quail’s comfort behaviours (STI, scratching: control quail: 53%, treated quail: 44%, *P* = 0.43; dust-bathing: control quail: 53%, treated quail: 56%, *P* = 0.57; CTI, scratching: control quail: 33%, treated quail: 55%, *P* = 0.27; dust-bathing: control quail: 25%, treated quail: 55%, *P* = 0.15).

### Spatial Learning Test

**Figure 2:**
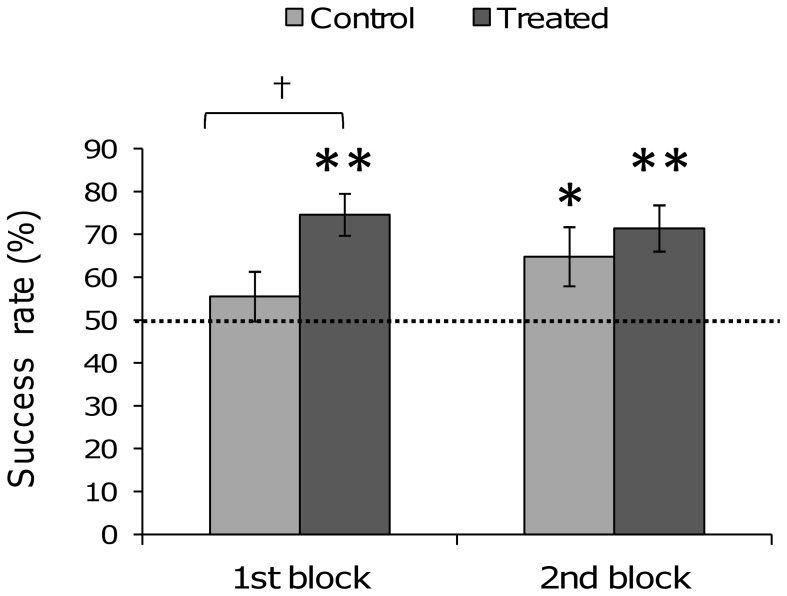
Spatial learning success rates of control and treated quail, for the first block of trials (1 to 3) and for the second block of trials (4 to 6). Student t test compared to 50%: *P<0.05, **P<0.01; ANOVA, treatment effect: † *P*<0.05.

Genetic background had no effect on the success rates for the first part of the test (ANOVA for repeated measurements, *F_[2,144]_* = 0.74, *P* = 0.48). However, treated quail’s success rates during the first three trials were significantly higher than those of control quail (ANOVA for repeated measurements, *F_[1,144]_* = 6.11, *P* = 0.01, fig. 2). Moreover, only the mean success rate of treated quail was significantly above 50%, the learning criterion (Student t-test, control quail: *t* = 0.96, *df* = 35, *P* = 0.34; treated quail: *t* = 4.82, *df* = 42, *P*<0.0001, fig. 2). By contrast, no treatment effects on success rate could be evidenced for the second block of trials (*F_[1,146]_* = 0.36, *P* = 0.45, fig. 2) as all the quail had then learnt the food location. Neither treatment nor genetic line influenced quail’s memorisation evaluated seven days later by the retrieval test (ANOVA, genetic line: *F*[*_2,71_*] = 0.40, *P* = 0.67; treatment: *F* *F*[*_1,71_* = 0.15, *P* = 0.70) and quail remembered the food location (72% of the quail were successful, Chi square test compared to chance: *χ^2^* = 7.94, *df* = 1, *P* = 0.005).

**Figure 3:**
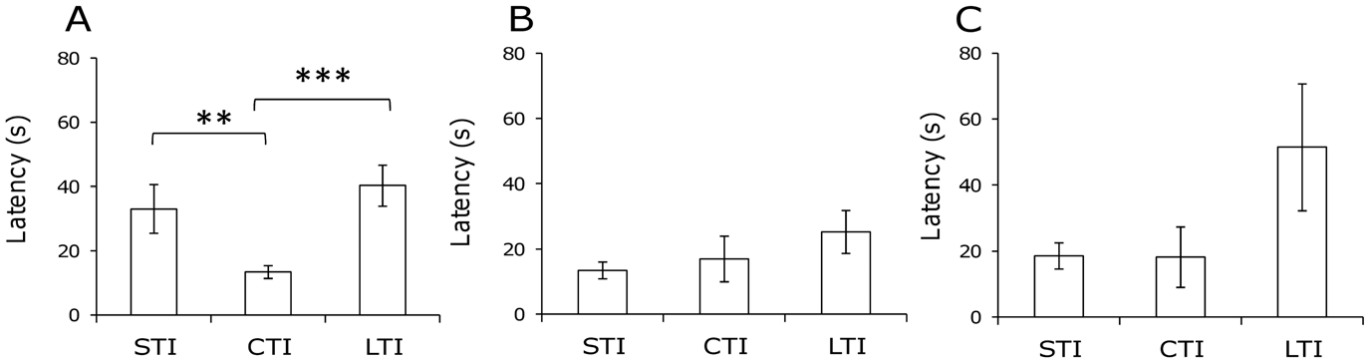
Mean latencies to reach the food in the spatial learning test for all quail from the three genetic lines, A: for the trial 1 to 3 (ANOVA, genetic line: *P* = 0.002, PLSD fisher test: **P<0.01, ***P<0.001), B: for trial 4 to 6 (ANOVA, *P*>0.05) and C: for the retrieval test (ANOVA, *P*>0.05).

Genetic line affected significantly latency to reach the rewarded trough during the first block of trials (ANOVA: genetic line, *F* *F*[*_2,71_* = 7.09, *P* = 0.002, fig. 3), but no differences between control and treated quail could be evidenced (*F* *F*[*_12,71_* = 0.53, *P* = 0.47). Post-hoc tests revealed that CTI quail reached the trough sooner than did either STI or LTI quail (fig. 3A). No significant effects were evidenced for the second block of trials (4 to 6) (ANOVA, genetic line: *F* *F*[*_2,71_* = 1.35, *P* = 0.26; treatment: *F* *F*[*_1,71_* = 0.006, *P* = 0.94, fig. 3B) or for the retrieval test (ANOVA, genetic line: *F* *F*[*_2,71_* = 2.25, *P* = 0.11; treatment: *F* *F*[*_1,71_* = 0.03, *P* = 0.87, fig. 3C).

## Discussion

This study shows that two weeks of chronic stress have several impacts on quail’s behaviour, evident both at the end of the procedure and several weeks later. Indeed, at the end of the procedure, stressed quail preened and rested more. After the procedure, they expressed higher emotional reactivity than did control quail. Finally, the stress procedure appeared to enhance spatial learning. However, intrinsic emotional reactivity had less effect than expected.

### Chronic Stress Effects on Homecage Activity

Spontaneous activity evaluated at the end of the chronic stress procedure differed significantly between treated and control quail, but not between genetic lines. Dalm et al. [59] pointed out a lack of studies concerning the detailed pattern of activity of rodent models of chronic stress in their home cage, and none of the few studies on bird’s chronic psychological stress evaluated behaviour in their home cage [3], [17], [18], [20]–[22], [60]. Our present study evidences that treated quail rested less than did control quail. This could be linked to several factors. First, treated quails were disturbed at least once during the dark phase and this increase of the time spent sleeping during the light phase could be a compensatory effect. Second, rest and sleep are strongly associated with energy conservation [61], [62] and this increase of the time spent resting could be linked to disturbed homeostasis, and thus energy consumption, due to the chronic stress procedure. Moreover, treated quail’s preening frequency increased. An increase of the time birds spend preening can indicate poor welfare [63]. Duncan and Wood-Gush [64] found that thwarting poultry’s feeding behaviour increased their displacement preening. This preening has been described as shorter than normal and relatively directed at specific parts of the body (e.g. neck and chest). Finally, treated quail’s foraging activity was reduced. This result can be compared to the alteration of the circadian activity in chronically stressed mice (*Mus musculus*), which spent less time near the feeder and the drinker [59].

All in all, these behavioural modifications presented by female Japanese quail in their familiar environment led us to conclude that our CSP induced chronic stress effects. However, further observations are needed to highlight these results. Indeed, although the effects of chronic stress on sleep and energy consumption have already been studied in humans and mice, this is not the case for birds. This should be considered in a further study as it would have direct implications for the management of poultry species in stock breeding.

### Chronic Stress Effects on Behavioural Development

#### A. Emotional reactivity

After two weeks of chronic stress during development, subsequent exposure to stressful situations revealed differences between stressed and control quail. The effects of the treatment on emotional reactivity depended both on the test conditions and on the quail’s intrinsic emotional reactivity. Actually, the behaviours of the least fearful (STI) control and treated quail differed neither in the emergence test, nor in the open field test. The most fearful (LTI) treated quail never displayed comfort behaviours in the open field arena whereas LTI control quail did, and they also explored less, revealing a higher level of emotional reactivity in novel environments [65]. Finally, stressed quail from the control genetic line (CTI) tested in a closed novel environment vocalized sooner and more frequently, indicating a higher level of emotional reactivity in response to social isolation than that of control CTI quail [56]. Moreover, they displayed more fear behaviours. All in all, our results show that intrinsic emotional reactivity modulates the sensitivity to the CSP. Whereas quail with medium or high levels of intrinsic emotional reactivity were affected by our treatment, it was not the case for the least fearful quail. Thus, as we hypothesized, intrinsic emotional reactivity modulated the sensitivity to CSP. Interestingly, Houdelier and collaborators [66] showed that STI quail chicks were also the least affected by the intrinsic fearfulness of their foster mothers. Indeed, whereas the ontogeny of emotional reactivity in CTI and LTI chicks was strongly influenced by their foster mothers’ behaviour, STI chicks’ behavioural development appeared to be unaffected by these maternal influences. Our results are also concordant with data for HPA axis activity in these two genetic lines. Indeed, LTI quail’s corticosterone baseline was lowered by repeated negative stressing stimulations, whereas STI quail’s HPA axis was not affected by this treatment [22]. STI and LTI quail’s autonomic responses to a startling stimulus also differed: LTI quail showed strong sympathetic activation, whereas both STI quail’s parasympathetic and sympathetic systems were activated [67]. Here we show that the effects of a two-week chronic stress procedure differed between genetic lines selected on tonic immobility that is to say with different levels of intrinsic fearfulness. Our treatment did not increase fearfulness in the ‘low reactive’ quail but increased the other lines’ fearfulness. Nonetheless, LTI quail did not seem to be more affected by the CSP than were CTI quail, contrary to what we expected. This result could be due to our procedure. Indeed, the aim of our stress protocol was not to induce very strong effects as in rodents that are generally used as a model for studying human depression [49], although a more intensive stress procedure might reveal more inter- and intra-strain variability, as in mice [68].

#### B. Spatial learning and memory

Our treatment appeared to enhance spatial learning. The first block of trials showed that only treated quail remembered the food location and their success rates were higher than those of control quail. During the second block of trials, success rates of control and treated quail no longer differed and all the quail had learnt the location of the food in the maze. All quail remembered the food location during the retrieval test, seven days after the last trial and the CSP did not affect their memorization.

Previously, chronic stress has been shown to impair cognitive abilities and especially spatial learning, in relation to a loss of dendritic morphology in specific brain regions like the hippocampus and prefrontal cortex [69], [70]. However, those protocols often implied severe and prolonged stressors like several hours of restraint [71] or social defeat followed by isolation [72], [73], often contingent with the learning process. On the contrary, mild or acute stressors can enhance cognitive abilities [12], [74]–[76]. Here, we suggest that treated quail’s higher emotional reactivity may have resulted in higher attention, thus enhancing their performance in the maze. This relationship has been widely studied in humans [77]–[79] and has also been artificially tested in rodents and birds [80], [81]. Injections of catecholamine, a physiological substrate of emotional arousal, increased the subjects’ attention and thus enhanced their learning and memory performances.

Second, the effect of CSP on learning performance in the T-maze could be partly explained by a modulatory effect of stress on memory consolidation or storage. Stressors occurring during the learning task alter memory consolidation [15], but after treatment, stressful events enhance the retention of information [74]. However, to modulate the memory system these emotions have to occur close enough in time to the learning task [74], [82]. For example, social isolation of domestic chicks just after passive avoidance training had the same effect as a corticosterone injection, resulting in an enhancement of retention [83]. Rats (*Rattus norvegicus*) tested in a Morris water maze in cold water in order to induce a moderate stress learn quicker and remembered better the platform location than did rats tested in warm water [84]. However, when these rats were injected with metyrapone, blocking the increase of the corticosterone level due to stress, their performance was no better in cold water than in warm water. Thus, as chronic stress enhances emotional reactivity, it could be responsible for better or faster acquisition in a spatial learning task. Sandi and Pinelo-Nava [27] proposed an inverted U-shaped relationship between stress intensity and spatial learning performance. If very high stress impairs spatial learning through deleterious effects on the hippocampus, on the contrary, medium to high stress could enhance memorisation of a spatial task. The fact that success rates of our treated quail were higher than those of control quail during the first part of training supports Sandi and Pinelo-Nava’s theory.

To our knowledge, we are the first to investigate learning capacities in adult quails selected on tonic immobility duration, a standard measure of fearfulness in birds. Surprisingly, quail’s intrinsic fearfulness did not modulate their success rates in the spatial learning task. However, their latencies to reach the rewarded trough differed, STI and LTI quail taking longer than CTI quail, especially at the beginning of the task. This result might reflect differences between the three lines concerning exploration and/or motivation to find the food, similar to the differences between foraging activity rates in the home cage. The lack of difference between genetic lines might be evidenced in involving other cognitive processes. Indeed, differences between strains of mice known to have different levels of susceptibility to chronic stress depend on the type of radial maze used [85]. C57BL/6 mice were the most successful in a task requiring spatial discrimination and also in the first part of a visual discrimination task, but at the end of the test, DBA/2 mice were as successful as C57BL/6 mice. Female rats were tested in three different memory tasks [75]. The performance of stressed females was enhanced in spatial tasks, but no differences could be evidenced between stressed and control females in an object recognition task. In quail submitted to one week of chronic stress, the two groups of females did not differ during acquisition but stressed females were more successful during the reversal training than were control females [21]. Therefore, the effects of our protocol should also be tested by different cognitive tasks to specify how different cognitive systems are modulated by the CSP.

### Conclusions

Our present study shows that a 2-week stress protocol modified Japanese quail’s behavioural development. We evidenced an increase of emotional reactivity that was modulated by the quail’s intrinsic characteristics, linked to their inherent fear determined by selection for tonic immobility duration. The quail’s learning performances were enhanced by chronic stress, concordantly with the inverted U-shaped theoretical relationship between stress and cognitive performances and highlighting the importance of emotional arousal in cognitive challenges. This study provides new fundamental knowledge about the effects of chronic stress on behaviour, until now mainly studied in mammals. These results are also relevant for applied ethology, to improve the welfare of captive animals, especially in stock breeding.
